# Current status and prospects of congenital adrenal hyperplasia: A bibliometric and visualization study

**DOI:** 10.1097/MD.0000000000040297

**Published:** 2024-11-08

**Authors:** Sheng Chen, Lingling Wu, Xiaohan Ma, Lin Guo, Jianqiang Zhang, Hongjun Gao, Ting Zhang

**Affiliations:** a Graduate School, Guangxi University of Chinese Medicine, Nanning, Guangxi, China; b Ruikang Hospital, Guangxi University of Chinese Medicine, Nanning, Guangxi, China.

**Keywords:** 21-hydroxylase deficiency, bibliometrics, citespace, congenital adrenal hyperplasia, Vosviewer

## Abstract

**Background:**

Congenital adrenal hyperplasia (CAH) is increasingly prevalent, leading to a surge in related research. To pinpoint emerging trends and recommend future directions, a bibliometric analysis of relevant CAH literature was performed.

**Methods:**

From January 1, 2000, to October 1, 2023, we searched the Web of Science Core Collection for CAH literature. For the bibliometric analysis, tools such as VOSviewer, CiteSpace, and the R package “bibliometrix” were employed.

**Results:**

The United States and England are at the forefront among 113 countries, contributing 5034 papers to CAH research. However, there is a need for more extensive global collaboration across institutions in this field. The number of publications on CAH is increasing annually. Leading research institutions include the University of Michigan, University of California, San Francisco, Karolinska Institutet, and Karolinska University Hospital. The Journal of Clinical Endocrinology & Metabolism is the most cited in this area. The most prolific author is Falhammar H, with 88 publications, 2568 co-citations, and a significant overall contribution. Key research areas include diagnostic methods and therapeutic strategies for CAH. Emerging research hotspots are identified by keywords such as “disorders of sex development,” “21-hydroxylase deficiency,” “ambiguous genitalia,” “testosterone” and “adrenal insufficiency.”

**Conclusions:**

Research on CAH is expected to expand globally. Future studies will primarily focus on exploring CAH’s diagnostic aspects and developing new therapies. This paper will help scholars better understand the dynamic evolution of the CAH and point out the direction for future research.

## 1. Introduction

A set of autosomal recessive disorders known as congenital adrenal hyperplasia (CAH) are brought on by mutations in genes that encode the enzymes necessary for the synthesis of adrenal steroids.^[1,2]^ The incidence of classic CAH, primarily caused by 21-hydroxylase deficiency (21OHD), varies by region and ethnicity.^[3]^ Non-classic CAH has a significantly higher incidence than its classic counterpart, particularly in highly consanguineous ethnic groups such as Ashkenazi Jews.^[4]^ To effectively treat classic CAH, 3 primary clinical goals must be balanced: managing iatrogenic hypercortisolism and its potential comorbidities; minimizing exposure to adrenal androgens at age- and sex-appropriate levels; and facilitating physiological replacement of adrenal insufficiency.^[5]^ Recent studies have made significant advancements in understanding the epidemiology, pathophysiology, treatment, and prevention of CAH. However, bibliometric analyses in this field are currently lacking. By using bibliometric methodologies, this study seeks to give a thorough picture of the knowledge structure and research hotspots in CAH.

Bibliometric analysis is the quantitative evaluation and study of all knowledge carriers in a certain topic using specialized statistical methodologies. This methodology facilitates the identification of research hotspots, the comprehension of the knowledge structure, the investigation of cooperative modalities, and the prediction of future development directions.^[6,7]^ This technique is widely used across various domains. Our paper aims to provide a summary of CAH literature characteristics, explore findings, and potential future research directions in this area, while also offering resources for further study.^[8]^

## 2. Materials and methods

### 2.1. Procedure

Bibliographic data was collected from the Web of Science Science Citation Index-Expanded database. To avoid discrepancies due to daily updates, we accessed the Web of Science Core Collection on October 2, 2023, and downloaded papers published between 1990 and 2023. We used search terms such as “CAH,” “Adrenogenital Syndrome,” “Hyperplasia, Congenital Adrenal,” “Adrenal Hyperplasias, Congenital,” “21OHD,” “11β-Hydroxylase Deficiency,” “17α-Hydroxylase Deficiency,” “3β-Hydroxysteroid Dehydrogenase Deficiency,” “P450 Oxidoreductase Deficiency,” and “Congenital Lipoid Adrenal Hyperplasia.” The inclusion criteria encompassed original research, reviews, and meta-analyses related to CAH. Excluded were conference abstracts, proceedings, case reports, correspondence, unpublished papers lacking detail for further study, and duplicates. The titles, keywords, authors, countries or regions, journals, institutions, and references of each article were collated and exported as a plain text file. Since this study is bibliometric and did not involve human subjects or animals, ethical approval was not required.

### 2.2. Data analysis

An innovative bibliometric analysis tool, VOSviewer (1.6.17), extracts key information from a wide range of publications.^[9]^ The software primarily conducts analysis in our study, including co-occurrence of authors, institutions, nations, and keywords. Network visualization, overlay visualization, and density visualization are the three modules that are included in this. CiteSpace (6.1.R1), a program mainly used for bibliometric analysis and visualization.^[10]^ We used CiteSpace for burst word and burst references analysis in our study. Each time slice was set to last 1 year, and the chosen time span stretched from January 2000 to October 2023. The R package “bibliometrix” (3.2.1) was used to construct a global distribution network of publications related to CAH.^[11]^

## 3. Results

### 3.1. Annual publications

**Figure 1.**
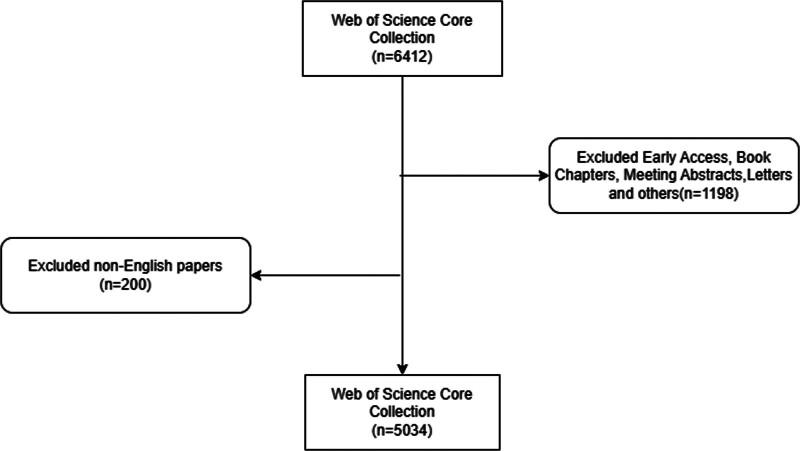
Flow diagram of the included papers.

**Figure 2.**
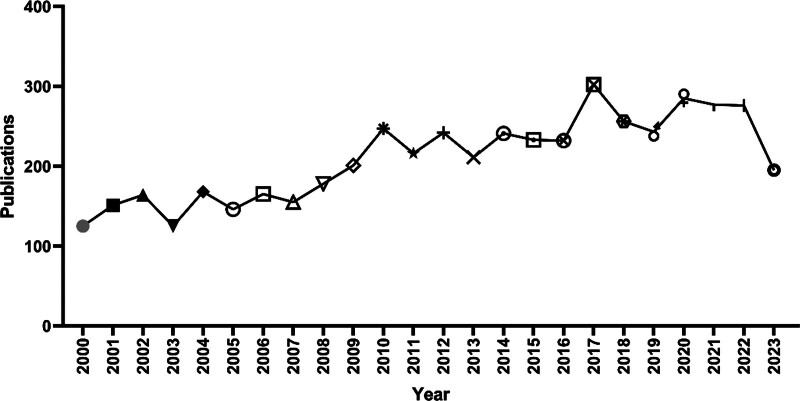
Trend of annual publishing volume between 2000 and 2023.

This analysis encompassed a total of 5034 articles (Fig. 1). A line graph, constructed using statistical analysis of the annual publication count (Fig. 2), revealed 4 peaks in the publication volume for CAH research in 2008, 2010, 2017, and 2020. This indicates increased research activity in these years. The decline in the number of papers published in 2023 can be attributed to the search cutoff point. In the past 5 years, the annual publication count has remained relatively stable, suggesting that CAH continues to be a significant and actively researched topic.

### 3.2. Analysis of leading journals

CAH-related articles have been published in a total of 1188 academic journals. Table 1 displays the top 10 journals, along with the number of articles each published. Leading the list is the Journal of Clinical Endocrinology & Metabolism with 339 articles, followed by the European Journal of Endocrinology (110 articles), Clinical Endocrinology (136 articles), and the Journal of Pediatric Endocrinology & Metabolism (182 articles). Additionally, the most cited journal among the top 10 is the Journal of Clinical Endocrinology & Metabolism, with 20,219 citations, followed by the European Journal of Endocrinology (3988 citations), Clinical Endocrinology (3666 citations), and the Journal of Steroid Biochemistry and Molecular Biology (2411 citations). These findings indicate the significant academic impact of these journals in the CAH field.

**Table 1:** The top 10 productive and cited journals on the research of CAH.

Rank	Journal	Publications	Count of citations
1	Journal of Clinical Endocrinology & Metabolism	339	20219
2	Journal of Pediatric Endocrinology & Metabolism	182	1935
3	Clinical Endocrinology	136	3666
4	European Journal of Endocrinology	110	3988
5	Hormone Research in Paediatrics	97	1767
6	Journal of Steroid Biochemistry and Molecular Biology	83	2411
7	Hormone Research	76	1963
8	Frontiers in Endocrinology	75	449
9	Journal of Pediatric Urology	60	788
10	Journal of Endocrinological Investigation	58	664

CAH = congenital adrenal hyperplasia.

### 3.3. Analysis of leading countries, regions, and institutions

**Figure 3.**
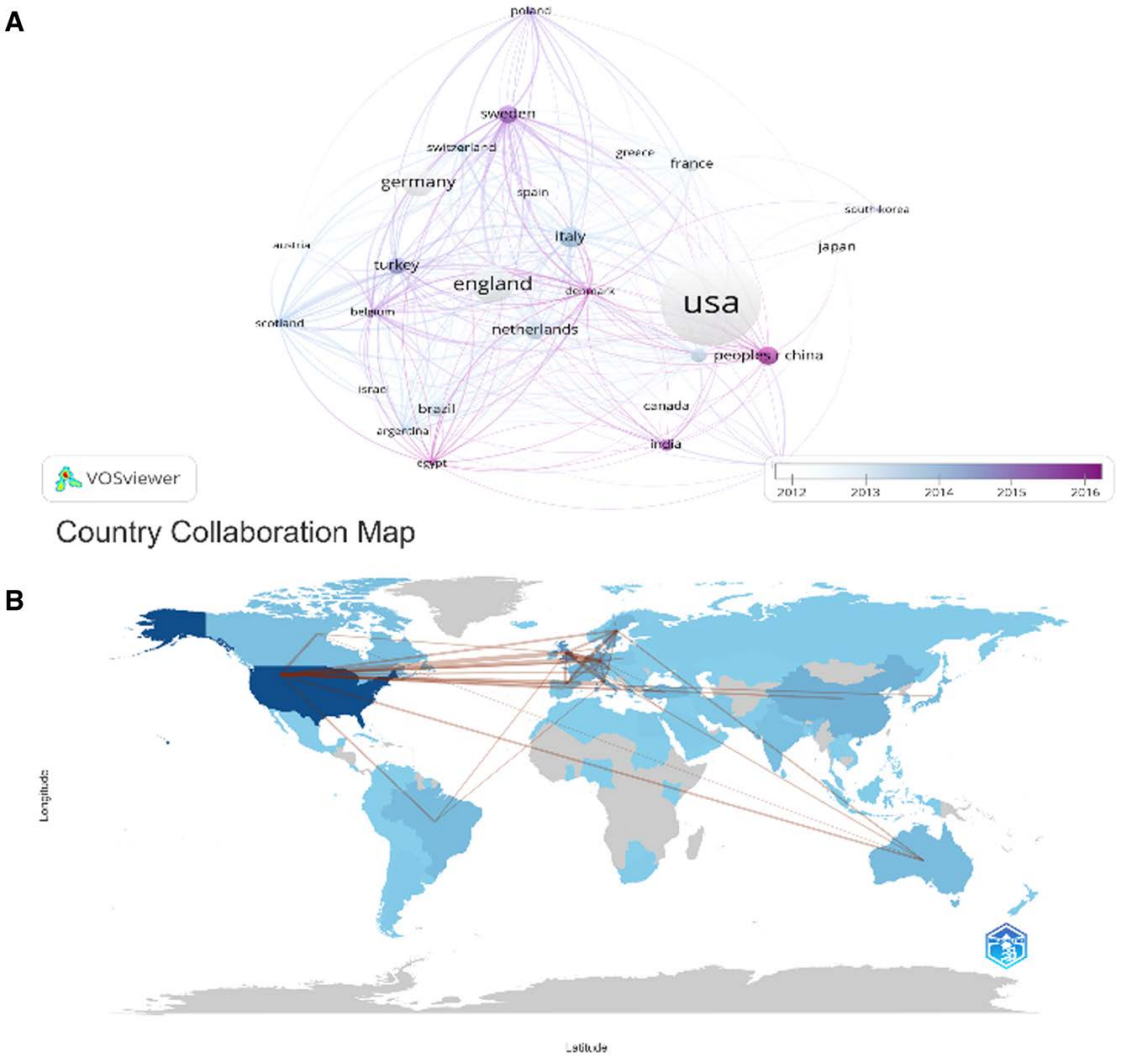
The visualization of countries (A) and geographical distribution (B) on CAH. CAH = congenital adrenal hyperplasia.

Our analysis of national and regional cooperative network maps for CAH-related research revealed the top ten contributing nations or regions (Table 2; Fig. 3). The United States leads with 1508 publications, followed by England (568 articles), Germany (414 articles), Italy (296 articles), and China (257 articles). The number of articles published in each nation is represented by the size of the circle in the network maps. Different hues indicate different eras, and the degree of cooperation is shown by the thickness of the lines. This shows that research on CAH has been conducted to differing degrees in various parts of the globe. The United States of America, England, Germany, the Netherlands, and Canada have the strongest collaborative ties. This suggests that there is an increasing trend of cross-national and cross-regional collaboration in CAH research.

**Table 2:** The top 10 productive countries/region on the research of CAH.

Ranking	Countries/region	Publications	Link strength
1	USA	1508	353
2	England	568	371
3	Germany	414	302
4	Italy	296	195
5	China	257	50
6	Netherlands	250	244
7	Sweden	239	224
8	Turkey	227	94
9	France	214	161
10	Brazil	203	102

CAH = congenital adrenal hyperplasia.

**Figure 4.**
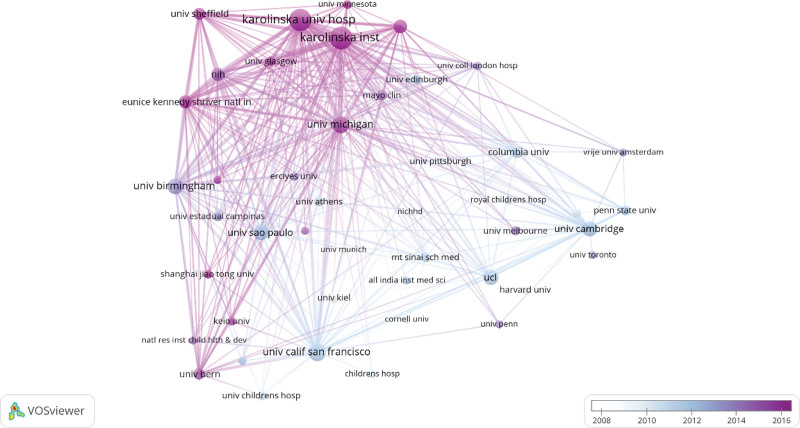
The network map of institutions.

Table 3 shows that the leading contributors to CAH research are the University of California, San Francisco (103 publications), Karolinska Institutet (168 publications), and Karolinska University Hospital (157 publications). The strength of institutional cooperation connections and the institutional network collaboration map (Fig. 4) highlight the need for increased cross-institutional collaboration in CAH research worldwide.

**Table 3:** The top 10 productive institutions on the research of CAH.

Ranking	Institutions	Publications	Link Strength
1	Karolinska Institutet	168	166
2	Karolinska University Hospital	157	160
3	University of California, San Francisco	103	28
4	University of Michigan	101	41
5	University of Birmingham	91	48
6	University of Sao Paulo	83	11
7	University of Cambridge	83	21
8	University College London	83	25
9	Radboud University Nijmegen	69	45
10	National Institutes of Health	64	77

CAH = congenital adrenal hyperplasia.

### 3.4. Analysis of leading authors and co-cited authors

**Figure 5.**
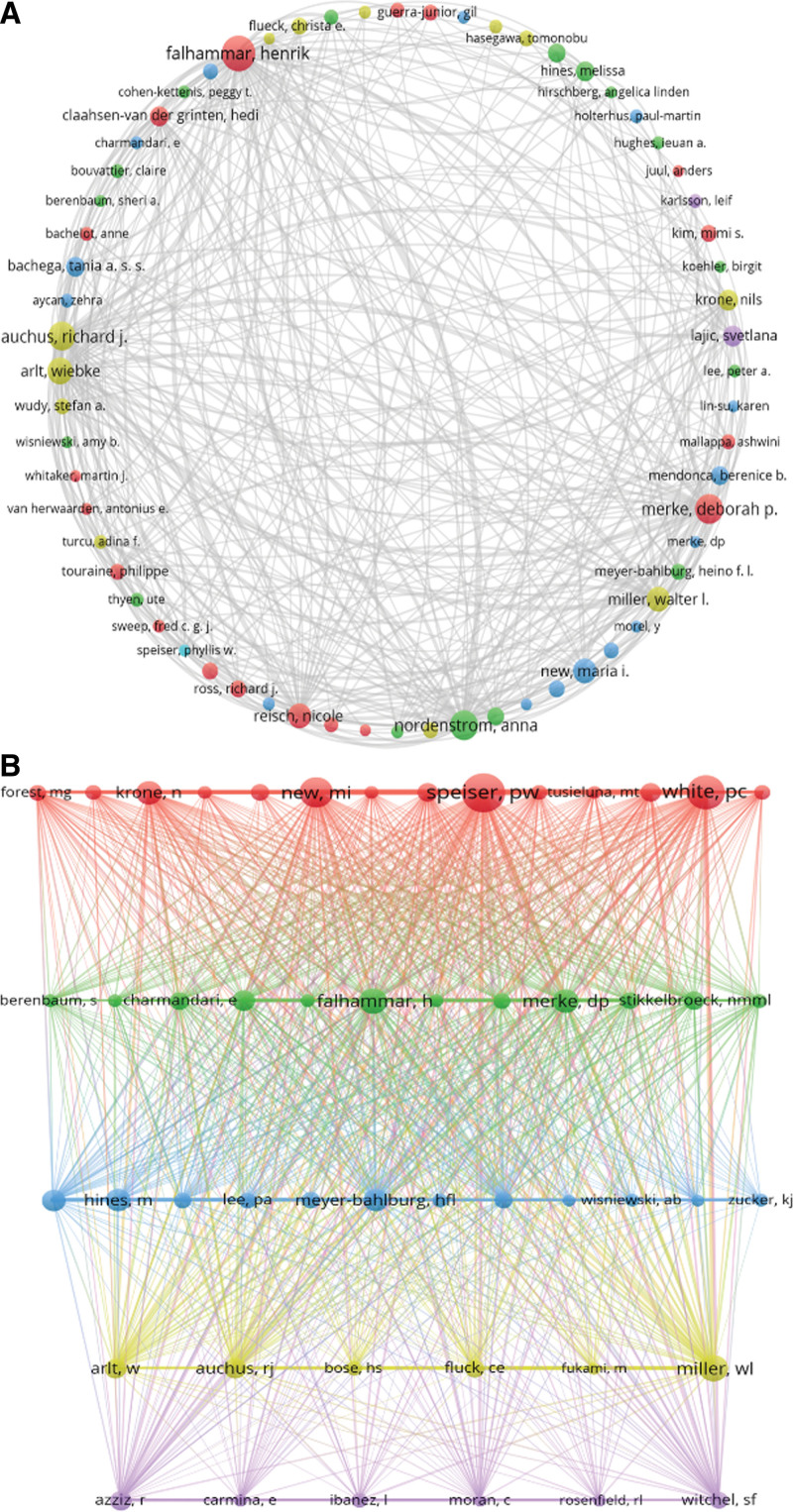
Cooperation map of authors.

The co-occurrence map of the author cooperation network (Fig. 5A) uses various colored circles to represent different clusters, with lines indicating the level of cooperation within these clusters. This research identifies three main author collaboration groups and a significant amount of author cooperation. Table 4 lists the top five authors with the most published documents: Falhammar H (88 articles), Merke DP (68 articles), Nordenström A (66 articles), Auchus RJ (64 articles), and Arlt W (56 articles). Among the 65,417 co-cited authors, 6 authors were co-cited over 1000 times, as detailed in Table 4.

**Table 4:** The top 10 productive and cited authors on the research of CAH.

Ranking	Author	Documents	Co-cited author	Count
1	Falhammar H	88	Speiser PW	2568
2	Merke DP	68	White PC	1999
3	Nordenström A	66	New MI	1576
4	Auchus RJ	64	Miller WL	1204
5	Arlt W	56	Falhammar H	1160
6	Miller WL	50	Merke DP	1008
7	Reisch N	50	Krone N	976
8	New MI	48	Hines M	928
9	Krone N	37	Meyer-Bahlburg HFL	914
10	Lajic S	36	Berenbaum SA	841

CAH = congenital adrenal hyperplasia.

The most co-cited author in our study is Speiser PW (2568 co-citations), followed by White PC (1999 co-citations) and New MI (1576 co-citations). To map the co-citation network graphs (Fig. 5B), authors with at least 250 co-citations were included. As depicted in Figure 5B, active collaborations are also evident among different co-cited authors, such as between Falhammar H and Merke DP, and Nordenström A and Auchus RJ.

### 3.5. Analysis of keywords and burst words

**Figure 6.**
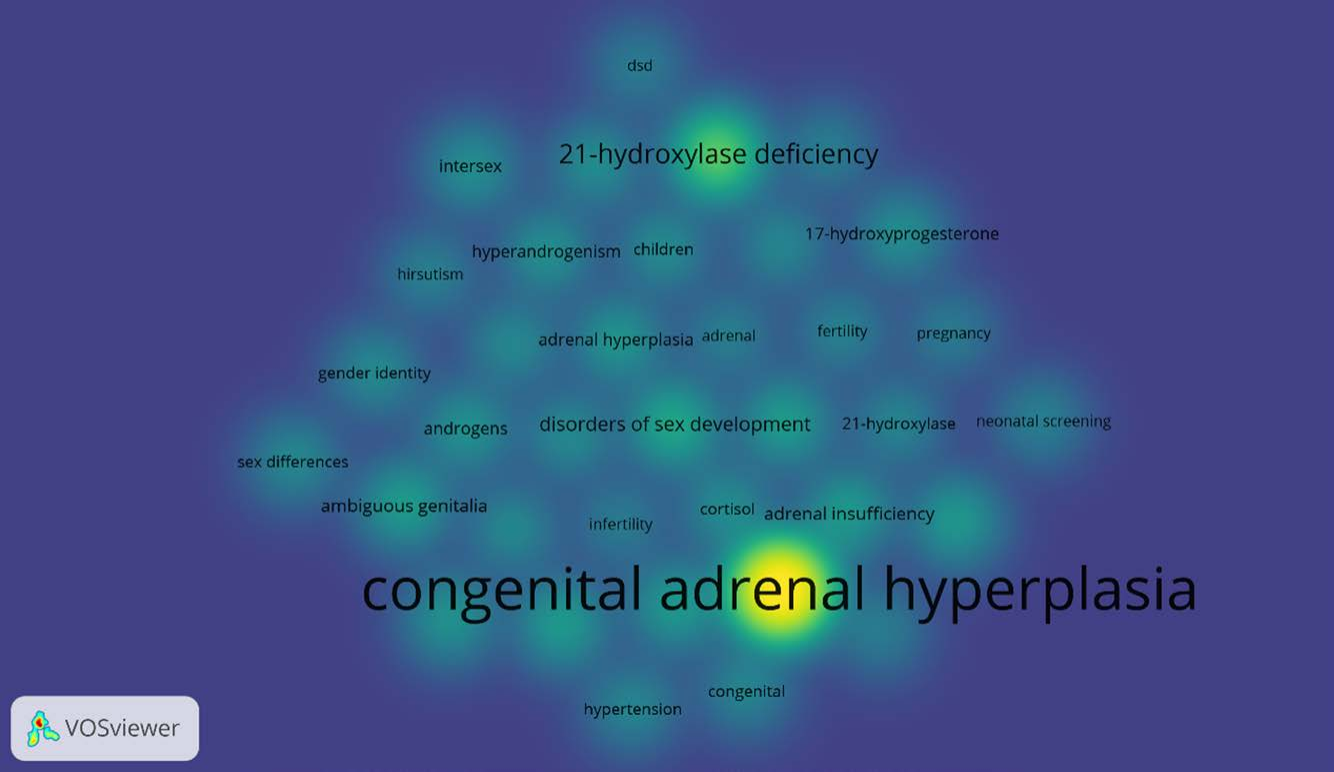
Keyword co-occurrence map of publications on CAH research. CAH = congenital adrenal hyperplasia.

Research hotspots and evolving trends can be discerned by analyzing keywords, which encapsulate and extract the main ideas of articles. The frequency of these keywords is closely linked to the level of interest in the corresponding research topics.^[12]^ In this study, we analyzed a total of 36 keywords with a minimum occurrence frequency of 45. Prominent keywords included “disorders of sex development” (159 occurrences), “ambiguous genitalia” (122 occurrences), “adrenal insufficiency” (115 occurrences), “CAH” (1356 occurrences), and “21OHD” (337 occurrences). The research hotspots encompassed the fields of basic medicine, clinical medicine, and epidemiology, as depicted in Figure 6.

**Figure 7.**
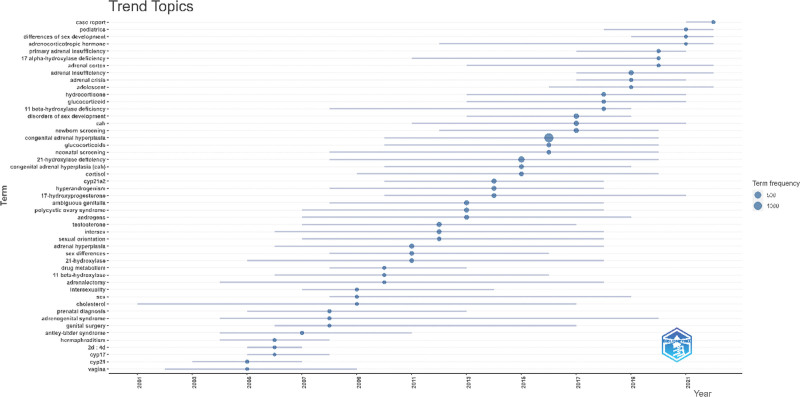
Trend topics on CAH research. CAH = congenital adrenal hyperplasia.

We also used the R software’s bibliometrix package to create a trend topic map (Fig. 7). This map shows the historical development of certain research themes within that field. We were able to follow the development and focal points of each stage of the CAH study by looking at the trend topic map in Figure 7. Our results show that the majority of the research being done in this area focuses on diagnosing and treating CAH.

**Figure 8.**
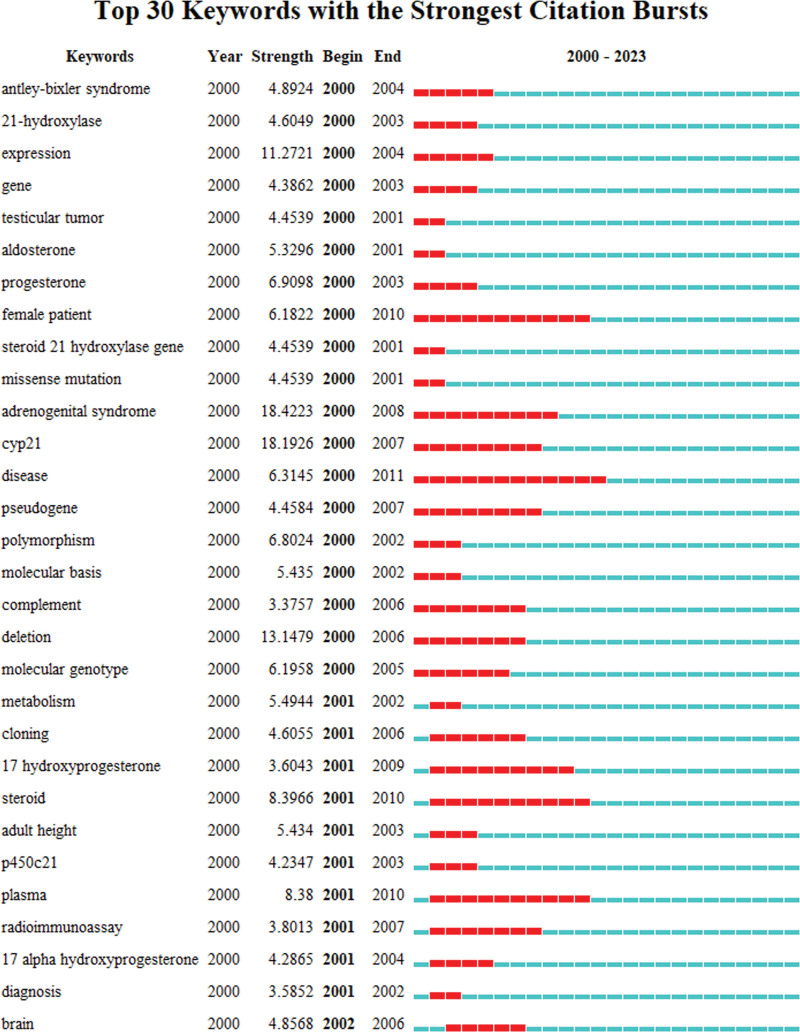
Top 30 keywords with the strongest citation bursts.

The term “burst word” refers to a term whose frequency significantly rises within a brief period. These words often signal research hotspots, allowing us to deduce overarching trends and directions in research based on their emergence and decline.^[13]^ In this study, the focus of burst words shifted from identifying CAH disease to exploring its mechanisms, as illustrated in Figure 8.

### 3.6. Co-cited references and reference with citation bursts

**Figure 9.**
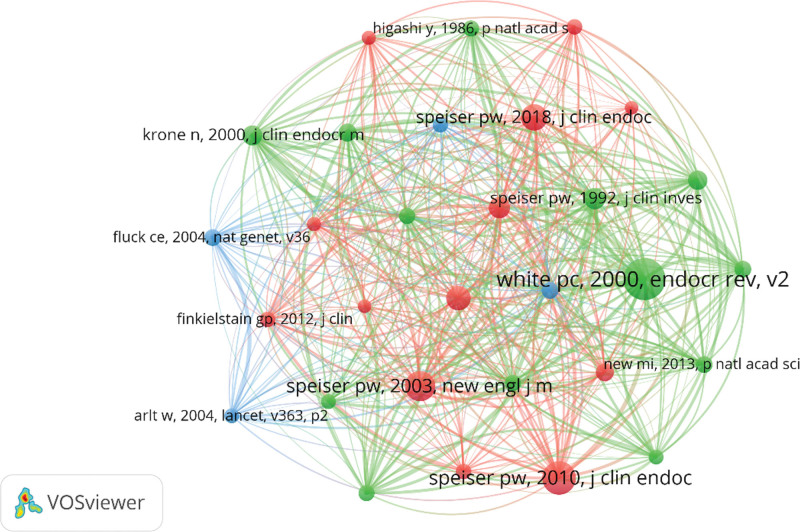
The visualization of co-cited references on research of CAH. CAH = congenital adrenal hyperplasia.

The study conducted by CAH has accumulated 105,211 co-cited references in the last thirty years. At least 228 citations have been made to each of the top 10 references (Table 5), with the most cited reference having nearly 773 citations. For constructing the co-citation network map (Fig. 9), we included references with 154 or more co-citations. Figure 9 highlights the prominent co-cited connections, especially between “Krone N, 2000, J Clin Endocrinol Metab” and “White PC, 2000, Endocr Rev,” as well as “Speiser PW, 2003, N Engl J Med,” and “Speiser PW, 2018, J Clin Endocrinol Metab,” among others.

**Table 5:** Top 10 co-cited references on research of CAH.

Ranking	Co-cited reference	Citations
1	White PC, 2000, Endocr Rev, v21, p245	773
2	Speiser PW, 2010, J Clin Endocrinol Metab, v95, p4133	563
3	Speiser PW, 2003, N Engl J Med, v349, p776	480
4	Speiser PW, 2018, J Clin Endocrinol Metab, v103	396
5	Merke DP, 2005, Lancet, v365, p2125	354
6	Speiser PW, 1992, J Clin Invest, v90, p584	292
7	Arlt W, 2010, J Clin Endocrinol Metab, v95, p5110	283
8	Krone N, 2000, J Clin Endocrinol Metab, v85, p1059	265
9	Speiser PW, 1985, Am J Hum Genet, v37, p650	251
10	Wedell A, 1994, J Clin Endocrinol Metab, v78, p1145	228

CAH = congenital adrenal hyperplasia.

**Figure 10.**
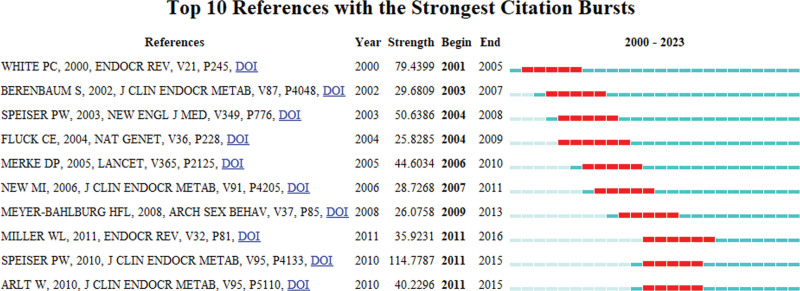
Top 10 references with strong citation bursts. A red bar indicates high citations in that year.

References experiencing citation bursts are those frequently cited in a particular field over a specific period. In our study, CiteSpace identified 10 references with significant citation bursts (Fig. 10). As depicted in Figure 10, each bar corresponds to a year, with red bars indicating periods of intense citation activity.^[14]^ Citation bursts for references in our study spanned from as early as 2001 to as late as 2011. The most strongly cited reference, with a burst strength of 114.77, was “CAH Due to Steroid 21OHD: An Endocrine Society Clinical Practice Guideline” by Phyllis W. Speiser et al, showing citation bursts from 2011 to 2015. The second-highest burst (strength = 79.43) was observed in “CAH due to 21OHD” by White PC, et al, published in Endocrine Reviews, with bursts from 2001 to 2005. The burst strengths of these 10 references ranged from 25.82 to 114.77, with durations spanning 4 to 5 years. Table 6 provides a summary of the main research topics of these 10 references, ordered as they appear in Figure 10.

**Table 6:** The main research contents of the 10 references with strong citations bursts.

Rank	Strength	Main research content
1	79.43	Treatment of CAH focuses on hormone replacement and early detection to minimize risks.^[42]^
2	29.68	The guidelines advocate for early diagnosis, optimal therapy, and patient adherence, highlighting the importance of multidisciplinary care from childhood through adulthood. They also identify existing knowledge gaps and underscore the necessity for further research into innovative treatments.^[43]^
3	50.63	The molecular mechanism, diagnosis and treatment of CAH are introduced.^[44]^
4	25.82	The involvement of Cytochrome P450 oxidoreductase in drug metabolism has enabled linking Antley–Bixler syndrome and amenorrhea to fluconazole consumption in mothers with Cytochrome P450 oxidoreductase mutations, an enzyme crucial for the function of specific other enzymes.^[45]^
5	44.60	In its most severe form, individuals also exhibit adrenal medulla anomalies and a deficiency of adrenaline.^[46]^
6	28.72	Administering dexamethasone effectively reverses symptoms and manifestations of hyperandrogenism.^[47]^
7	26.07	Most women in the study identified as heterosexual. However, both nonclassical and classical CAH women showed higher rates of bisexual and homosexual orientation compared to controls. This trend was also associated with the degree of prenatal androgenization.^[48]^
8	35.92	The process of steroidogenesis, which involves converting cholesterol into steroid hormones, encompasses transport proteins, enzymes, redox partners, and cofactors.^[49]^
9	114.77	It is recommended to screen newborns for severe steroid-21OHD.^[50]^
10	40.22	The study found that androgen levels were poorly regulated and glucocorticoid replacement often nonphysiological in the subjects. This correlated with a poor metabolic profile, reduced quality of life, and fertility issues. Improving clinical care for individuals with CAH is essential.^[51]^

## 4. Discussion

### 4.1. General information

The adrenal gland has significantly shaped the history of pediatric endocrinology. The 19th century’s discovery of CAH highlighted the adrenal gland’s role in influencing sexual phenotypes and its vital importance for survival. In the early 20th century, the extraction of various adrenal steroids led to the distinction between androgens, mineralocorticoids, and glucocorticoids through bioassays. The 1950s marked a pivotal era with Wilkins, Bartter, and Albright’s groundbreaking cortisone treatment for CAH, propelling pediatric adrenal research. Wilkins’ thorough clinical research established the modern CAH treatment protocol. In 1957, Alfred Bongiovanni identified impaired 21-hydroxylation in CAH, followed by discoveries of deficits in 11β-hydroxylase and 3β-hydroxysteroid dehydrogenase. The first activity attributed to a P450, 21-hydroxylation, was characterized between 1962 and 1964. Accurate measurement of 17OH-progesterone levels in infants and their response to adrenocorticotropic hormone (ACTH) facilitated the identification of CAH in children and their families. From 1984 to 2004, molecular genetics techniques unraveled the genetic and metabolic underpinnings of various diseases.^[15]^

The prevalence of CAH has led to more stringent guidelines for its diagnosis, management, and treatment, prompting an increase in research activity. Recent years have seen a steady output of around 250 publications annually. In contrast, from 2000 to 2003, the field was nascent, with fewer than 150 papers published yearly, indicating a lack of a robust scientific foundation. The period from 2009 to 2013 marked an early stage in CAH research, with an average of 223 papers published annually. However, there was a significant uptick in research output from 2016 to 2020, with an average of 263 papers per year. The last 3 years have seen a further increase in relevant publications, suggesting a growth phase in CAH research. Currently, the field is expanding, attracting more researchers to related studies.

CAH research is dominated by the United States and England, with the US being at the forefront. The United States has produced 1508 papers, followed by England with 568, and Germany with 414. Among the top ten research institutions, the US and the UK are most prominent. Collaboration is notable between the US, UK, Canada, and Germany, with Germany also maintaining substantial collaborations with Spain, France, and Italy. However, the extent and level of cooperation among institutions are not ideal. For example, there is limited collaboration between Chinese and Polish institutions, which could hinder long-term academic progress. Therefore, we advocate for research institutions across national boundaries to engage in extensive cooperation and communication to collectively further the development of CAH research.

Journal of Clinical Endocrinology & Metabolism (IF = 5.80) publishes most CAH research, making it the most prominent journal in this field. The Lancet, with an impact factor of 168.90, ranks as the highest-impact journal, followed by the New England Journal of Medicine (IF = 158.50). Our analysis reveals that most co-cited journals are high-impact Q1 journals. These publications, which are instrumental in supporting CAH research, are undoubtedly among the world’s top-tier periodicals.

Nordenström A, Merke DP, and Falhammar H are the most prolific authors in CAH research. Falhammar H has authored 88 publications, 5 of which delve into the diagnosis, management, and risk factors of nonclassical CAH.^[16–20]^ A notable study by Falhammar H associates poor therapy adherence rates, rather than the type of glucocorticoid, with reduced quality of life in adults and adolescents with CAH.^[21]^ Merke DP, with 68 publications, contributed significantly to understanding CAH’s link to various comorbidities, including hypogonadism, and suggested that enlarged adrenal glands and increased testosterone synthesis could lead to metabolic risk factors.^[22]^ The author of 66 publications, Nordenström A, noted that fractures linked to osteoporosis are more common in CAH patients of both sexes, but not in those screened as babies.^[23]^ Collectively, these authors focus on the pathophysiology, diagnosis, and management of CAH.

Speiser PW, with 2568 citations, is the most frequently co-cited author in this field, followed by White PC (1999 citations) and New MI (1576 citations). According to White PC’s 2011 study, hyperkalemia does not always mean that a kid has primary adrenal insufficiency. The study emphasized that adrenal insufficiency should be considered in cases of hypotension, hyponatremia, and chronic or subacute symptoms.^[24]^ A 2014 study suggested that massively parallel sequencing of 3.6 mL of plasma from pregnant women could noninvasively detect CAH before the 9th week of pregnancy.^[25]^ Speiser PW discovered regional variations in CAH newborn screening practices in the US in 2020.^[26]^

### 4.2. Hotspots and frontiers

Research hotspots and future directions in the field of CAH are summed up by combining the frequency of occurrence of keywords with Citation Bursts and burst word mapping.

#### 4.2.1. Diagnosis of CAH

Steroid hormone and precursor level measurement is fundamental in diagnosing and managing CAH. Presently, the assessment of steroid hormones utilizes analytical methods based on immunoassay techniques or chromatographic methods combined with mass spectrometry.^[27]^

A salt-losing CAH evaluation should be conducted immediately on infants with ambiguous genitalia, bilateral non-palpable gonads, and positive results from newborn screening. Progesterone, androstenedione, 17-hydroxyprogesterone, and plasma renin activity should all be measured as well as electrolyte concentrations. Elevated 17-hydroxyprogesterone levels, usually >5000 ng/dL in afflicted neonates, corroborate the diagnosis of 21-hydroxylase insufficiency.^[28]^ For virilized females, chromosome tests and pelvic ultrasounds are advised to confirm a XX karyotype and uterus presence.^[29]^ An ACTH stimulation test may be necessary for definitive CAH evaluation in any age group. A cortisol response to ACTH exceeding 18 mcg/dL indicates normal hypothalamus–pituitary–adrenal axis activity.^[30]^ Cortisol evaluation is crucial to assess cortisol secretion adequacy, particularly in individuals with Nonclassical Congenital Adrenal Hyperplasia.^[31]^ Prenatal diagnosis is feasible when both parents are carriers of CYP21A2 mutations, typically when they already have a child with 21OHD. Over recent decades, the range of available prenatal diagnostic methods has expanded. However, invasive sampling should be reserved for cases where the results would alter management or treatment strategies.^[32]^ Preimplantation genetic diagnosis is offered in many countries for families at risk of severe genetic conditions like 21OHD.^[33]^ For preimplantation genetic diagnosis, a biopsy of the blastocyst trophectoderm on days 5 to 6.^[32]^

#### 4.2.2. Treatment of CAH

The primary aim of treating classic CAH is to supplement glucocorticoid and mineralocorticoid hormones.^[2]^ During the neonatal period, to rapidly lower elevated androgen levels, some clinicians administer higher than recommended doses of glucocorticoids. However, this approach necessitates more frequent monitoring to swiftly adjust the dosage upon achieving target steroid levels, thereby avoiding the adverse effects of high glucocorticoid doses.^[34]^ Hydrocortisone (HC), the synthetic form of cortisol, is preferred for growing children with classic CAH due to its shorter half-life, which helps minimize side effects, especially growth suppression, often seen with longer-acting, more potent glucocorticoids.^[2]^ Hormonal control during puberty can be challenging, even with adequate replacement doses and high medication adherence. This is partly due to decreased activity of 11-beta-hydroxysteroid dehydrogenase 1, affecting HC’s pharmacokinetics. Consequently, higher glucocorticoid doses may be required during puberty.^[35]^

In treating adults with classic CAH, the choice of glucocorticoid depends on the physician’s clinical experience and the patient’s individual needs. Immediate-release HC is the preferred glucocorticoid treatment for adults.^[36,37]^ Most girls born with classic CAH present with virilized external genitalia, which may include fused outer labia, a single opening of a common urogenital sinus, a recessed vagina entering the common channel, and clitoromegaly.^[38]^ Feminizing surgery is often performed in early childhood to create a female genital appearance.^[39]^ Surgical techniques have evolved to maintain clitoral sensitivity while minimizing vaginal stenosis.^[40]^

## 5. Strengths and limitations

The strength of this study lies in its bibliometric analysis of the global research landscape of CAH, offering valuable insights into authors, journals, institutions, research hotspots, and frontiers for relevant researchers. However, a limitation is the exclusive analysis of the Web of Science Core Collection database, chosen for its perceived value and reliability. This focus excluded meetings, books, and other types of publications from the data collection, resulting in a limited number of articles being considered.^[41]^

## 6. Conclusion

Over the past three decades, CAH research has seen significant growth. Studies published in specialized journals have garnered more attention than those in general journals. Enhancing collaboration between countries, institutions, and authors can further advance this field. CAH remains a promising area of study, where advancements in treatment and management are crucial for improving patient quality of life. Given current trends, global research on CAH is poised to expand, with future efforts likely concentrating on deeper exploration of its mechanisms and the development of new treatment methods and targets.

## Author contributions

**Conceptualization:** Sheng Chen, Lingling Wu, Ting Zhang.

**Data curation:** Sheng Chen, Lingling Wu, Xiaohan Ma.

**Formal analysis:** Lingling Wu, Lin Guo, Hongjun Gao.

**Funding acquisition:** Lingling Wu, Hongjun Gao.

**Investigation:** Lingling Wu, Ting Zhang.

**Methodology:** Lingling Wu, Xiaohan Ma, Lin Guo.

**Project administration:** Jianqiang Zhang.

**Resources:** Hongjun Gao, Ting Zhang.

**Software:** Lin Guo.

**Supervision:** Lingling Wu.

**Validation:** Sheng Chen, Lingling Wu.

**Visualization:** Sheng Chen, Jianqiang Zhang.

**Writing – original draft:** Sheng Chen, Lingling Wu, Jianqiang Zhang.

**Writing – review & editing:** Lingling Wu, Jianqiang Zhang, Hongjun Gao.
